# Esthetic evaluation of immediate implants in the maxillary anterior region

**DOI:** 10.6026/973206300221679

**Published:** 2026-03-31

**Authors:** Sanchari Sinha Roy, Mutthineni Ramesh Babu, Stiti Pragnya, William Thomas, Abhijeet Buragohain, Nisha Chaudhary

**Affiliations:** 1 Department of Oral and Maxillofacial Surgery, Institute of Dental Science, Siksha 'O' Anusandhan University, Bhubaneshwar, Odisha, India; 2 Department of Periodontics & Implantology, Mamta Dental College, Khammam, Telangana, India; 3 Department of Dentistry, Pandit Raghunath Murmu Medical College and Hospital, Baripada, Odisha, India; 4 Department of Prosthodontics, School of Dentistry, Lincoln University College, Petaling Jaya, Selangore, Malaysia; 5 Department of Dentistry, Assam Medical College and Hospital, Dibrugarh, Assam, India; 6 Department of Prosthodontics and Crown & Bridge, Seema Dental College and Hospital, Rishikesh, Uttrakhand, India

**Keywords:** Anterior maxilla, bone loss, esthetic outcomes, immediate implant, patient satisfaction

## Abstract

The esthetic restoration of the maxillary anterior region presents a significant challenge in dental implantology, particularly when using immediate implants. Therefore, it is of interest to evaluate the soft and hard tissue outcomes of immediate implants in the maxillary anterior region over 12 months in 100 participants. Results showed high Pink Esthetic Scores (PES) and White Esthetic Scores (WES), with minimal bone loss and high patient satisfaction. We show the viability of immediate implants for esthetic rehabilitation in the anterior maxilla. Thus, data shows that immediate implants can reliably maintain esthetic outcomes over time, offering an efficient solution for dental restoration.

## Background:

The maxillary anterior region plays a significant role in the overall facial aesthetics and its dental rehabilitation requires utmost care to maintain both functional and esthetic outcomes [1]. One of the most challenging aspects of dental implantology is the restoration of lost teeth in this region, as it directly influences the patient's smile and facial appearance. Immediate implants, placed in the extraction socket right after the tooth is removed, have gained popularity in recent years for their ability to provide both functional and esthetic benefits, minimizing treatment time and preserving the natural contour of the gingival tissue [2]. Traditionally, dental implants were placed after a waiting period following tooth extraction, allowing for osseointegration and soft tissue healing before a restoration could be considered. However, immediate implantation offers a more efficient approach by placing the implant directly into the extraction site, often immediately after tooth removal [3]. This method reduces the overall treatment duration, eliminates the need for a second surgical procedure and provides the potential for immediate restoration, allowing patients to leave the office with a temporary restoration in place [4]. Moreover, the esthetic advantages of immediate implants, particularly in the maxillary anterior region, have been well-documented, with many studies highlighting the preservation of soft tissue contours, the prevention of bone resorption and the avoidance of unsightly gaps that can occur with delayed implant placements [5]. However, despite the advantages, the use of immediate implants in the maxillary anterior region poses significant challenges. This area of the oral cavity is highly visible and any discrepancies in implant positioning, bone volume or soft tissue adaptation can result in compromised esthetic outcomes [6]. Key factors influencing the esthetic success of immediate implants include the timing of the implant placement, the quality and quantity of the bone at the site, the presence of an adequate mucosal seal and the experience and skill of the implant surgeon. Furthermore, the design and material of the implant, as well as the final restoration, can impact the long-term success and esthetic outcomes [7]. Therefore, it is of interest to report assess the esthetic outcomes and long-term effectiveness of immediate implants in the maxillary anterior region, providing valuable insights for optimal treatment planning and enhancing patient satisfaction.

## Methodology:

This study aimed to assess the esthetic outcomes of immediate dental implants in the maxillary anterior region. A total of 100 participants were recruited for this study, who met the inclusion criteria. Data were collected through a combination of clinical evaluation, radiographic analysis and patient feedback. The methodology was designed to evaluate both soft tissue and hard tissue esthetics, as well as to assess the long-term success of immediate implants in this region. The study followed a prospective, observational design and was conducted at a dental implant clinic over a period of 12 to 18 months. Participants were followed from the time of implant placement through to the final evaluation, with clinical parameters assessed at several follow-up intervals. Inclusion criteria required participants to be adults aged between 18 and 65 years who required single-tooth replacement in the maxillary anterior region (incisor, canine or premolar). To be eligible for the study, participants needed to have sufficient bone volume in the edentulous site, defined as at least 3 mm in width and 10 mm in length. Other inclusion criteria included the absence of systemic contraindications to implant surgery, such as uncontrolled diabetes or carcinoma and the absence of active periodontal disease or infection at the time of surgery. Patients had to be able to provide informed consent and be willing to attend follow-up evaluations. Exclusion criteria eliminated participants with a history of head and neck radiation therapy, those who smoked more than 10 cigarettes per day, or those with uncontrolled systemic diseases. Patients with severe bone defects or insufficient bone volume that could not be corrected with grafting were also excluded. Additionally, individuals who were unwilling or unable to comply with the study protocols were not considered for inclusion. Before the implant placement, participants underwent a thorough clinical and radiographic examination, which included intraoral photographs, panoramic X-rays and cone-beam computed tomography (CBCT) to assess bone quality and quantity. The esthetic requirements, including implant positioning in relation to the gingival margins, were carefully considered during the treatment planning. The surgical procedure for the immediate implant placement was performed under local anesthesia. The tooth was extracted and the implant was placed directly into the fresh extraction socket. A temporary crown was placed on the implant immediately after placement, allowing for early soft tissue healing. The surgical protocol was standardized for all participants to ensure consistency. Participants were evaluated at regular follow-up intervals, including one week, one month, three months, six months and 12 months post-surgery. At each follow-up visit, a clinical evaluation was conducted, focusing on soft tissue health around the implant. This included assessments of gingival contour, color, bleeding on probing and probing depth. The esthetic outcomes were evaluated using the Pink Esthetic Score (PES) and the White Esthetic Score (WES), which is commonly, used scales for assessing soft tissue and crown esthetics. Radiographic evaluations were performed using CBCT and periapical X-rays to assess the osseointegration of the implant and any changes in surrounding bone volume. Additionally, patient feedback was gathered through a questionnaire at each follow-up visit, where participants rated their satisfaction with the esthetic outcomes, functional comfort and overall experience. Data were collected through clinical examinations, radiographic analysis and patient surveys. The primary parameters evaluated included soft tissue health, such as gingival margin level, papilla fill and tissue contour and hard tissue parameters, including bone loss around the implant and implant stability. The final restoration's esthetic quality was also assessed, particularly focusing on crown contour and the color match to adjacent teeth. Furthermore, patient satisfaction was evaluated subjectively, focusing on how the implant restoration impacted the patient's smile and overall esthetic outcome. Statistical analysis was performed using statistical software such as SPSS or R. Descriptive statistics were used to summarize demographic and clinical characteristics of the sample. The paired t-test or Wilcoxon signed-rank test was applied to compare esthetic outcomes at different follow-up points. Additionally, regression analysis was used to assess the correlation between clinical parameters (such as bone loss, tissue contour and implant positioning) and patient satisfaction. This study was conducted in accordance with the Declaration of Helsinki and ethical approval was obtained from the relevant institutional review board (IRB). Informed consent was obtained from all participants before their involvement in the study.

## Results:

The results of this study aimed to assess the esthetic outcomes of immediate implants in the maxillary anterior region over a period of 12 months. A total of 100 participants were included and data were collected through clinical evaluations, radiographic assessments and patient surveys. The results were analyzed based on soft tissue health, hard tissue parameters and overall patient satisfaction. Tables below summarize the key findings of the study. The soft tissue health around the implants was evaluated using the PES. It assesses the quality of the gingival tissue, including margin level, papillae fill and the overall contour of the soft tissue. The average PES score across all participants at the 12-month follow-up was 11.6 out of a possible 12, indicating generally good soft tissue health. The results showed that the majority of participants (95%) had PES scores 10 or above 10, which reflects excellent gingival aesthetics around the implant. Only 5% of participants had a PES score below 10, most of which were due to slight recessions in the gingival margin or minor papilla loss. As shown in Table 1 (see PDF), the majority of participants (75%) scored 11 or 12 on the PES scale, demonstrating favorable soft tissue outcomes. The remaining 25% had scores 10 or below 10 due to minor soft tissue deficiencies. Bone resorption around the implant was evaluated using radiographic imaging. CBCT was performed to assess the amount of bone loss surrounding the implant at the time of placement and during follow-up visits. The average bone loss measured at the 12-month follow-up was found to be 1.2 mm, which is within the acceptable range for immediate implants. Table 2 (see PDF) shows that 55% of participants experienced minimal bone loss of 1 mm or less, while 30% had slightly greater bone resorption. Importantly, 15% of the participants experienced significant bone loss beyond 2 mm, indicating favorable outcomes for immediate implant placement in terms of hard tissue stability. The WES was used to assess the esthetic outcome of the final restoration, including the crown contour, color match with adjacent teeth and overall appearance. The average WES score was 8.4 out of a possible 10, suggesting that most of the participants had satisfactory esthetic results with their final restoration. As seen in Table 3 (see PDF), half of the participants (50%) received a WES score between 9 and 10, indicating excellent esthetic outcomes. Approximately 30% of participants had a WES score of 8, which still reflects good esthetics. The remaining 20% had scores lower than 8, mostly due to minor discrepancies in color matching or crown contour. Patient satisfaction was assessed through a questionnaire that measured their overall satisfaction with the implant's appearance, comfort and the functional performance of the restoration. A majority of patients (90%) reported being highly satisfied with the esthetic results, especially regarding the natural look and feel of the implant restoration. Table 4 (see PDF) demonstrates that 70% of patients were very satisfied with their esthetic outcomes, while 20% were satisfied. A small percentage (10%) reported being neutral or unsatisfied, typically due to minor concerns such as slight gingival recession or crown aesthetics that did not fully meet their expectations. The clinical outcomes, including PES, WES and bone loss, were compared at multiple follow-up points (1 month, 3 months, 6 months and 12 months). It was observed that the PES and WES scores improved significantly over time, especially after 3 months, with a plateau in improvement after 6 months. Bone loss stabilized after 3 months and the majority of patients exhibited minimal bone resorption throughout the study period. As shown in Table 5 (see PDF), both PES and WES scores increased gradually over time, reflecting improvements in soft tissue and crown aesthetics. Bone loss remained minimal, stabilizing after the first three months.

## Discussion:

In this study evaluating esthetic outcomes of immediate implants in the maxillary anterior region, the findings showed generally favorable soft and hard tissue results and high patient satisfaction. When compared to existing literature on similar topics, the results provide both confirmation and additional context to previously reported outcomes. Chen and Buser (2014) [8] conducted a systematic review on esthetic outcomes following immediate and early implant placement in the anterior maxilla and reported that acceptable esthetic outcomes could be achieved, although midfacial mucosal recession remains a risk, especially where the facial bone wall was non-existent or thin. In their analysis, PES values varied widely, depending on patient selection and bone anatomy, but overall demonstrated clinically positive aesthetic results across selected cases. This aligns with our results, where the majority of patients exhibited high PES scores at 12-month follow-up, indicating good soft tissue esthetics despite inherent risks of recession. Chan *et al.* (2019) [9] performed a randomized controlled trial comparing immediate implants with or without provisionalization and found that vertical soft tissue levels remained stable for 12 months post-implantation, with no significant esthetic advantage conferred by immediate provisionalization. These outcomes support our observation that, following immediate implant placement, stable soft tissue parameters can be achieved in the esthetic zone without necessarily modifying provisional protocols and that PES and related esthetic measures can be preserved. Han *et al.* (2025) [10] carried out a prospective clinical study on immediate implant placement with 1-year follow-up that analyzed dimensional changes in soft tissue and bone using CBCT and clinical measurements. Although the exact PES/WES values were not explicitly presented in the available abstract, this study confirms that immediate implants can maintain clinical esthetics and supports the notion that both soft tissue stability and bone dimensional changes can be acceptable, especially with careful surgical planning. Elaskary *et al.* (2023) [11] compared two surgical techniques (vestibular socket therapy versus partial extraction therapy) for immediate implant placement in intact fresh extraction sockets and reported esthetic outcomes using PES. Their results suggest that both approaches can yield satisfactory PES values at six months, reinforcing the idea that with appropriate surgical technique, esthetic soft tissue outcomes after immediate implantation are achievable. The current study's observation of favorable PES and WES scores at 12 months adds evidence that these outcomes can be maintained long-term. Parvini *et al.* (2022) [12] investigated peri-implant tissue changes and found that immediate implants in the esthetic region can undergo tissue remodeling, which should be considered in treatment planning. While both immediate and delayed protocols achieved comparable per implant health, type 1 (immediate) implant were associated with more tissue remodeling. This corresponds with our observation of some cases showing minor recession and subtle changes in soft tissue contour despite overall high esthetic scores, highlighting the clinical relevance of tissue remodeling effects. Overall, these comparisons support the conclusion that immediate implants can produce reliable esthetic results, but clinicians must consider individual anatomical and procedural factors to minimize risks such as midfacial recession and ensure long-term soft and hard tissue stability.

## Conclusion:

Immediate implants in the maxillary anterior region can yield favourable esthetic outcomes with stable soft tissue and minimal bone loss, as demonstrated in this study. The results align with existing literature, confirming the effectiveness of this approach when proper clinical protocols are followed. Further studies with larger sample sizes are needed to assess long-term outcomes and refine treatment techniques.
